# Identification of Metabolic Alterations in Breast Cancer Using Mass Spectrometry-Based Metabolomic Analysis

**DOI:** 10.3390/metabo10040170

**Published:** 2020-04-24

**Authors:** Sili Fan, Muhammad Shahid, Peng Jin, Arash Asher, Jayoung Kim

**Affiliations:** 1 West Coast Metabolomics Center, University of California, Davis, CA 95616, USA; slfan@ucdavis.edu; 2 Departments of Surgery, Cedars-Sinai Medical Center, Los Angeles, CA 90048, USA; muhammad.shahid@cshs.org (M.S.); Peng.Jin@cshs.org (P.J.); 3 Samuel Oschin Comprehensive Cancer Institute, Cedars-Sinai Medical Center, Los Angeles, CA 90048, USA; Arash.Asher@cshs.org; 4 Departments of Surgery and Biomedical Sciences, Cedars-Sinai Medical Center, Los Angeles, CA 90048, USA; 5 Department of Medicine, University of California, Los Angeles, CA 90095, USA; 6 Department of Urology, Ga Cheon University College of Medicine, Incheon 461-701, Korea

**Keywords:** biomarkers, mass spectrometry, metabolomics, breast cancer

## Abstract

Breast cancer (BC) is a major global health issue and remains the second leading cause of cancer-related death in women, contributing to approximately 41,760 deaths annually. BC is caused by a combination of genetic and environmental factors. Although various molecular diagnostic tools have been developed to improve diagnosis of BC in the clinical setting, better detection tools for earlier diagnosis can improve survival rates. Given that altered metabolism is a characteristic feature of BC, we aimed to understand the comparative metabolic differences between BC and healthy controls. Metabolomics, the study of metabolism, can provide incredible insight and create useful tools for identifying potential BC biomarkers. In this study, we applied two analytical mass spectrometry (MS) platforms, including hydrophilic interaction chromatography (HILIC) and gas chromatography (GC), to generate BC-associated metabolic profiles using breast tissue from BC patients. These metabolites were further analyzed to identify differentially expressed metabolites in BC and their associated metabolic networks. Additionally, Chemical Similarity Enrichment Analysis (ChemRICH), MetaMapp, and Metabolite Set Enrichment Analysis (MSEA) identified significantly enriched clusters and networks in BC tissues. Since metabolomic signatures hold significant promise in the clinical setting, more effort should be placed on validating potential BC biomarkers based on identifying altered metabolomes.

## 1. Introduction

Breast cancer (BC) is the most commonly diagnosed cancer in women in the U.S. and is the second leading cause of cancer-related death [1]. The American Cancer Society (ACS) predicts that there will be approximately 276,480 new diagnoses of invasive BC and 48,530 new cases of carcinoma in situ (CIS) BC in the U.S. in 2020. The ACS also estimates that 42,170 women will die from BC or BC-related complications in 2020. Early diagnosis of BC is crucial for a better prognosis. Currently, there are various ways of detecting BC, including breast exams and imaging techniques, such as mammography, magnetic resonance imaging (MRI), positron-emission tomography (PET), computed tomography (CT), and single-photo emission computed tomography (SPECT) [2]. However, these techniques have limitations, including cost, time, and suitability for different age groups [3]. In addition to these diagnostic methods, there are different BC markers that are being clinically used. These include tissue markers, such as hormone receptors, human epidermal growth factor-2, and urokinase plasminogen activator, genetic markers, such as breast cancer 1 (BRCA1) and BRCA2, and serum markers, such as CA 15.3 and BR 27.29 [4]. However, these markers are unable to diagnose BC in its early stages and can be affected by variations in disease state or therapies [5]. Therefore, there is an urgent need for a reliable rapid method of early BC detection.

Metabolomics involves studying the entire set of metabolites in a biological system, which is reflective of cellular functions and phenotypes [6]. It is well-documented that cancer cells display significantly altered cellular processes compared to normal cells, and these differences can be key to identifying a wealth of information, including cancer progression and therapeutic response [7]. One of the most established examples of cancer metabolic reprogramming is the “Warburg effect”, which leads to increased aerobic glycolysis in cells [8]. Metabolomics is a promising approach, particularly when it comes to BC, which is known to have complex and distinct molecular characteristics [9]. Metabolites are the ultimate results of downstream genes, RNA, and proteins, which allow for a better understanding of the complex biological interactions underlying BC [10]. By profiling the metabolic landscape of BC, novel molecular signatures can be identified that can not only provide early detection but also predict therapeutic response [11].

Alterations in amino acid transporters and glutamine metabolism have recently emerged as a field of interest in BC metabolomics [12]. Amino acids are the main molecules necessary for protein synthesis; as a result, growing tumor cells have an increased demand for them [13]. Several amino acid transporters, including SLC1A5, SLC6A14, and SLC7A5, were found to have varied expression in BC tissue compared to controls based on the type of cancer (i.e., HER2+ or ER+) [14,15,16]. Recent studies have also looked into how amino acid radiotracers can be utilized to better image BC [17]. One study demonstrated that both primary and metastatic sites of BC can be visualized via PET using 11C-methionine, a radioactively labeled amino acid [18]. Another study applied the same approach using 18F-fluciclovine, which found that uptake of the compound was 4-fold greater in malignant BC compared to normal [19]. This suggests that metabolic alterations can be used to diagnose BC, subtype the disease, and be applied in conjunction with other methods to more accurately characterize it in each patient.

There are a variety of metabolomic tools that can be applied when studying BC, including liquid chromatography-mass spectrometry (LC-MS), gas chromatography-MS (GC-MS), and nuclear magnetic resonance (NMR). Recent studies have even utilized ex vivo proton high-resolution magic angle spinning magnetic resonance (HR MAS MR) spectrometry on intact BC tissue samples to identify differentially expressed metabolites (DEMs), which found characterizable differences in levels of glycine, choline, and amino acid metabolism [20]. Based on these promising results, metabolomics is becoming more of an appealing and opportune avenue for future BC studies.

In this study, we aimed to identify metabolomic signatures capable of differentiating BC patients from healthy controls and to understand enriched metabolic pathways in BC. Two independent metabolomic profiling analyses found that glutamine levels were increased in BC. Further analysis using Chemical Similarity Enrichment Analysis (ChemRICH), MetaMapp, and Metabolite Set Enrichment Analysis (MSEA) found interesting BC-enriched clusters and networks.

## 2. Materials and Methods

### 2.1. Ethics Statement

The Institutional Review Board (IRB) at Cedars-Sinai Medical Center (CSMC) approved this study for metabolomics profiling and data analysis of BC samples collected through the CSMC Biobank’s central IRB (Pro00044997). All experiments were performed in accordance with relevant guidelines and regulations.

### 2.2. GCTOF MS Analysis

Sample extraction and gas chromatography-time-of-flight-mass spectrometry (GC-TOF-MS) analysis was performed as described in previous papers [21,22]. Briefly, tissue samples were prepared in conical polypropylene centrifuge tubes. Then, extraction solvent (acetonitrile, isopropanol, and water in a proportion ratio of 3:3:2) was added to the samples and homogenized for 45 s to ensure the uniform suspension of samples. After samples were centrifuged at 1500× g for 5 min, the supernatant was aliquoted into two 500 µL samples, one for analysis and another as backup. To process and analyse the supernatant, the samples were evaporated in a Labconco Centrivap Cold Trap Concentrator to complete dryness and resuspended in 500 µL of 50% acetonitrile. The samples were then centrifuged for 2 min at 14,000 rcf and the resulting supernatant was transferred to new Eppendorff tubes. This supernatant was again evaporated to complete dryness in the Labconco Centrivap Cold Trap Concentrator. For quality assurance, one blank negative control extraction was performed by applying the full procedure without any true biological samples for each sequence in the sample extraction.

#### 2.2.1. Data Acquisition

Data are acquired using the following chromatographic parameters as described with minor modifications [23]. Restek Rtx-5Sil MS Columns (30 m length × 0.25 mm internal diameter with 0.25 μm film made of 95% dimethyl/5%diphenylpolysiloxane) were used under the optimized running condition. The analytical GC column was protected by a 10 m long empty guard column, which was cut into 20 cm intervals whenever the reference mixture containing quality control (QC) samples indicated problems caused by column contaminations. This resulted in excellent retention and separation of primary metabolite classes (amino acids, hydroxyl acids, carbohydrates, sugar acids, sterols, aromatics, nucleosides, amines and miscellaneous compounds). Automatic liner exchanges, after each set of 10 injections, were done to reduce sample carryover for highly lipophilic compounds. The following MS parameters were used; Leco Pegasus IV Mass Spectrometer with a unit mass resolution at 17 spectra s^−1^ from 80–500 Da at −70 eV ionization energy and 1800 V detector voltage with a 230 °C transfer line and a 250 °C ion source.

#### 2.2.2. Data Processing

After data acquisition, raw data files were preprocessed using ChromaTOF vs. 2.32. Apex masses were reported for use in the BinBase algorithm and the resulting *.txt files containing absolute spectra intensities were stored in a data server for further processing. The BinBase algorithm (rtx5) was used with the following settings: validity of chromatogram (<10 peaks with intensity>10^7^ counts/second), unbiased retention index marker detection (MS similarity > 800, validity of intensity range for high *m*/*z* marker ions), and retention index calculation by 5th order polynomial regression. Spectra were cut to 5% base peak abundance and matched to database entries from most to least abundant spectra. The filters used included: retention index window ± 2000 units (equivalent to about ± 2 s retention time), validation of unique ions and apex masses (unique ion must be included in apex masses and present at >3% of base peak abundance), mass spectrum similarity must fit criteria dependent on peak purity and signal/noise ratios, and a final isomer filter. The failed spectra were placed into new database entries when s/n >25, purity <1.0 and presence in the biological study design class was >80%. The BinBase administration software, BinView, was also used for analysis. For each metabolite, the number of high-confidence peak detections and the ratio of the average height of replaced values to high-confidence peak detections were stored.

### 2.3. HILIC-ESI-QTOF-MS/MS Analysis

Samples extraction and hydrophilic interaction liquid chromatography-electrospray ionization quadruple time-of-flight tandem mass spectrometry (HILIC-ESI-QTOF-MS/MS) analysis was performed as described in previous papers [21,22].

#### 2.3.1. Data Acquisition

Data were acquired using the following chromatographic parameters, established as standard procedure in the Fiehn Laboratory. Analysis was done via HILIC-QTOF-MS/MS. The Waters Acquity UPLC BEH Amide Column (1.7 μm, 2.1 × 150 mm) and Waters Acquity and UPLC BEH Amide VanGuard Pre-Column (1.7 μm, 5 × 2.1 mm) were used at 40 °C and under a 0.4 mL/min flow rate. The injection volume was 3 μL for ESI (+), mass resolution was 10,000 for ESI (+) on an Agilent 6530 QTOF MS, and the scan range was *m*/*z* 60–1200 Da. The analytical ultra high-performance liquid chromatography (UHPLC) column was protected by a short guard column. This chromatography method yields excellent retention and separation of metabolite classes (biogenic amines, cationic compounds) and good within-series retention time reproducibility.

#### 2.3.2. Data Processing

The raw data were processed in an untargeted (qualitative) manner using mzMine 2.0 to find peaks in up to 300 chromatograms. Alternatively, selected peaks were collated and constrained into Agilent’s MassHunter quantification method on the accurate mass precursor ion level, using the MS/MS information and the NIST14 / Metlin / MassBank libraries to identify metabolites with manual confirmation of adduct ions and spectral scoring accuracy. MassHunter enables back-filling of quantifications for peaks that were missed in the primary peak finding process, hence yielding datasets without missing values. All metabolites were identified using the Fiehn library, which is publicly available at: http://massbank.us.

### 2.4. Bioinformatics Analysis for Identification of Metabolic Marker Candidates

To identify potential metabolites as marker candidates that can discriminate BC from controls, the following steps were applied. Data were normalized and the *t*-test was applied to the log2 of the processed data. The Student’s *t*-test was performed to extract significant metabolites from the normalized MS data. After false positive correction (FDR) using the Benjamini–Hochberg procedure, none of the p-values remained significant on the chosen level of 0.05.

Significant metabolites were selected for by the volcano plot based on a fold-change threshold > 2 (or < 0.5) and *t*-test *p*-value threshold < 0.1. MetaboAnalyst version 3.0. Log transformation and mean-centering with auto scaling was performed prior to multivariate statistical analysis. Partial least square discriminant analysis (PLS-DA) and model evaluation with permutation strategy was then carried out according to published protocols [23].

To further confirm potential metabolic markers in distinguishing BC from controls, a support vector machine (SVM)-based classifier was built using MATLAB (R2014a). Tenfold cross-validation was applied to evaluate performance. All annotated compounds were validated with authentic standards and are MSI level 1 [24,25].

### 2.5. Statistical Analysis

#### 2.5.1. Univariate Analysis

To identify significant compounds comparing BC vs. controls, the Mann–Whitney U test was performed on each compound. Benjamini–Hochberg FDR correction was utilized to deal with the multiple comparison problem. Fold-changes, defined as the median average of BC divided by the median average of the control samples, were calculated. Volcano plots visualizing the univariate analysis result for each identified metabolite with adjusted *p*-values less than 0.05 and fold-change greater than 2 or less than 0.5 were identified.

#### 2.5.2. Multivariate Analysis

**Figure 1:**
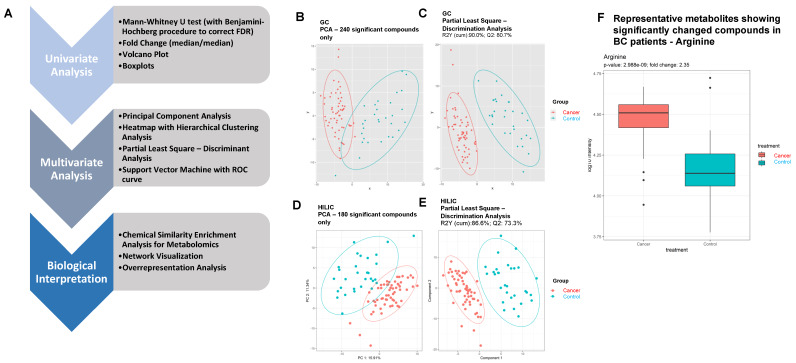
(**A**) Workflow for this study. (**B**) Principal component analysis (PCA) score plot visualization using 240 identified metabolites with Mann–Whitney U test raw *p*-values less than 0.05 in the GC platform. (**C**) Partial least square–discriminant analysis (PLS-DA) score plot of cancer vs. control. PLS-DA plot shows a clear separation of metabolites between BC patients and controls. Red: control samples; Green: BC patient samples. The model was established using three principal components. Cumulative R2 archived 90.0%, and Q2 achieved 80.7% with permutation test *p*-values less than 0.05. (**D**) PCA score plot visualization using 180 identified metabolites with Mann–Whitney U test raw *p*-values less than 0.05 in the HILIC platform. (**E**) PLS-DA score plot on cancer vs. control. Cumulative R2 archived 86.6%, and Q2 achieved 73.3% with permutation test *p*-values less than 0.05. (**F**) Boxplot of representative metabolite (arginine) showing significant alteration in BC patients.

Principal component analysis (PCA) using significant compounds are shown in Figure 1B. The 25 identified metabolites with the smallest *p*-values were used to construct the heatmap and hierarchical clustering analysis. Euclidean distance and Ward’s method were used. Two supervised learning algorithms, PLS-DA and SVM, were built. The Q2 was reported as a measure of the predictive performance of the PLS-DA model, whereas the cross-validated area under the curve (AUC) of the receiver operating characteristic (ROC) curve was the measure for the SVM algorithm. Permutation tests were used as validation for performance. The variable importance in projection (VIP) scores for compounds are reported, and VIP scores greater than 1 were considered as important.

### 2.6. Biological Interpretation

Chemical Similarity Enrichment Analysis for Metabolites (ChemRICH) was conducted to calculate metabolite cluster statistics based on chemical similarities. The Kolmogorov–Smirnov test was used on the identified clusters to evaluate whether a metabolite cluster was represented more than expected by chance. These clusters are visualized by bubble plot, where the bubble size indicates the number of metabolites in corresponding cluster and the color indicates the percentage of increased metabolites, with red being 100% and blue being 0%. To visualize the metabolite changes, MetaMapp was used. MetaMapp-encoded chemical structures of all the identified metabolites were retrieved from the PubChem Compound Database using compound identifiers and the NCBI Batch Entrez Utility. MetaMapp uses the Tanimoto chemical similarity co-efficient (range 0.0 to 1.0, where a high score means high similarity between two metabolites) to calculate the similarity among the encoded structures, which are decomposed into a substructure matrix defined by an 881 bit substructure key fingerprint (metamapp.fiehnlab.ucdavis.edu). A Tanimoto score threshold of 0.7 was used to define the similarity cut-off among metabolites. To calculate the metabolite cluster statistics based on the human metabolic pathway-associated metabolite sets, over representation analysis (ORA) was also conducted.

## 3. Results

### 3.1. Characteristics of the Study Subjects

BC was clinically diagnosed by physicians at Cedars-Sinai using standard operating procedures (SOPs). Surgical tissue samples were collected through an already established pipeline at the Cedars-Sinai Biobank. In total, 99 deidentified breast tissue samples were obtained (59 BC tumor tissues and 40 normal-adjacent BC tissues).

### 3.2. Primary Metabolomics Analyses Were Performed via ALEX-CIS-GC-TOF-MS and HILIC-ESI-QTOF-MS/MS

To profile the BC-specific metabolomes, two independent MS platforms were used. They were the automated liner exchange-cold injection system-gas chromatography-time-of-flight-mass spectrometry (ALEX-CIS-GC-TOF-MS) and HILIC-ESI-QTOF-MS/MS, both of which were combined with BinBase data processing. A total of 442 and 559 compounds were detected in the GC and HILIC platforms, respectively. A workflow of this study is shown in Figure 1A.

GC-TOF-MS identified 203 significant differentially expressed metabolites (DEMs), as shown in Supplementary Table S1 (Mann–Whitney U test raw *p*-values less than 0.05, fold-change > 2 or <0.5). PCA score plots visualized the results from PCA discrimination analysis (Figure 1B). PLS components showed differentiation of the BC samples (red) from controls (blue) with good separation and dispersion; the cumulative R2Y achieved 90.0%, and Q2 achieved 80.7% with permutation test *p*-values less than 0.05. (Figure 1C). Metabolomics profiling using HILIC-ESI QTOF MS/MS identified 139 compounds (Supplementary Table S2). Both the PCA score plot (Figure 1D) and PLS components showed great segregation of the BC group from controls. The cumulative R2Y was 86.6% and Q2 was 73.3% with permutation test *p*-values less than 0.05 (Figure 1E). A representative boxplot (arginine) shows the significantly changed compounds in BC patients compared to controls (Figure 1F).

### 3.3. Identification of DEMs in BC Patients

**Figure 2:**
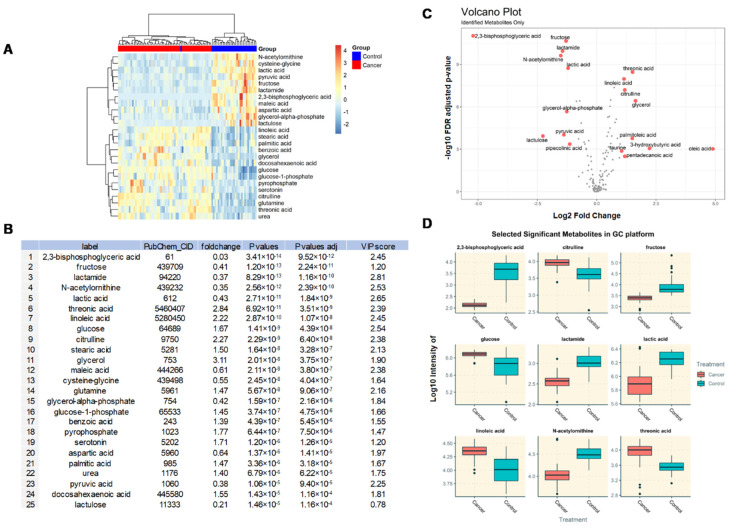
(**A**) Heatmap of identified metabolites in the GC platform showing the 25 differentially expressed metabolites (DEMs) between BC and control groups with the smallest Mann-Whitney U test *p*-values. Euclidean distance metric and Ward’s clustering method was used for the hierarchical clustering of samples and compounds. (**B**) A table summarizing the 25 identified metabolites and their statistical values. Fold-changes are presented as median average of cancer divided by control. False discovery rate-adjusted *p*-values using the Benjamini–Hochberg procedure are reported at the last column. VIP score for each compound is reported in Figure 3B (VIP > 1 highlighted as red). (**C**) Volcano plot with annotated metabolites that were significantly altered in BC patients compared to controls. The red dots represent metabolites above the threshold. The further the metabolite’s position away from the (0, 0), the more significant the metabolite is. Volcano plot visualizes the −log10 adjusted *p*-values and log2 fold-changes. Metabolites with adjusted *p*-values less than 0.05 and a fold-change greater than 2-fold or less than ½-fold are highlighted and labeled. (**D**) Selected boxplots of metabolites showing the most significant changes in the GC platform.

Among the 203 DEMs identified by GC-TOF-MS, only the top 25 DEMs, with fold-change > 1.20 or < 0.83 and *p*-values < 0.1 were selected and constructed into a heat map (Figure 2A). The heat map exhibits the distinct patterns of metabolites between BC and controls. These 25 DEMs are presented as log2 fold-changes against the −log10 (*p*) of the differential expression between BC and control samples. 

Significantly altered metabolites distinguishing cancer and control samples were acquired based on the Mann–Whitney U test *p*  <  0.05, fold-change > 2 or < 0.5, and VIP  >  1 (Figure 2B). A total of nine example metabolites were chosen for box-whisker plots (log10 scale) to visualize differences (Figure 2D). Outliers in these boxplots are defined as those greater than the 1.5 interquartile range (IQR). The volcano plots highlight metabolites with FDR *p*-values < 0.05 and fold-change > 1.5 or < 0.5. In total, there were 18 and 13 metabolites highlighted in the GC and HILIC platforms, respectively.

The levels of glycerol (fold-change of 3.10), glutamine (1.47), glucose-1-phosphate (1.44), benzoic acid (1.38), palmitic acid (1.47), urea (1.40), pyrophosphate (1.76), serotonin (1.70), docosahexaenoic acid (1.54) were significantly increased in the BC group compared to controls, with adjusted *p*-values < 0.001. In contrast, levels of 2,3-bisphosphoglyceric acid (fold-change of 0.027), fructose (0.40), lactamide (0.36), N-acetylornithine (0.34), lactic acid (0.43), maleic acid (0.60), cysteine-glycine (0.55), glycerol-alpha-phosphate (0.41), aspartic acid (0.63), pyruvic acid (0.38), and lactulose (0.20) were significantly decreased in the BC group compared to control, with adjusted *p*-values < 0.001 (Figure 2B).

A volcano plot (Figure 2C) and additional box plots (Figure 2D) show several representative DEMs whose expression were up- or downregulated in the BC group. The significant compounds with *p*-values less than 0.05 were used to conduct the PCA, with PC1 explaining 16% variance, and a PC2 of 11%. A clear separation between cancer and control samples are observed. The PLS-DA achieved a Q2 of 73.3%, with permutation test *p*-value less than 0.05, indicating a good predicting performance. The VIP score for each compound is reported in Figure 2B.

**Figure 3:**
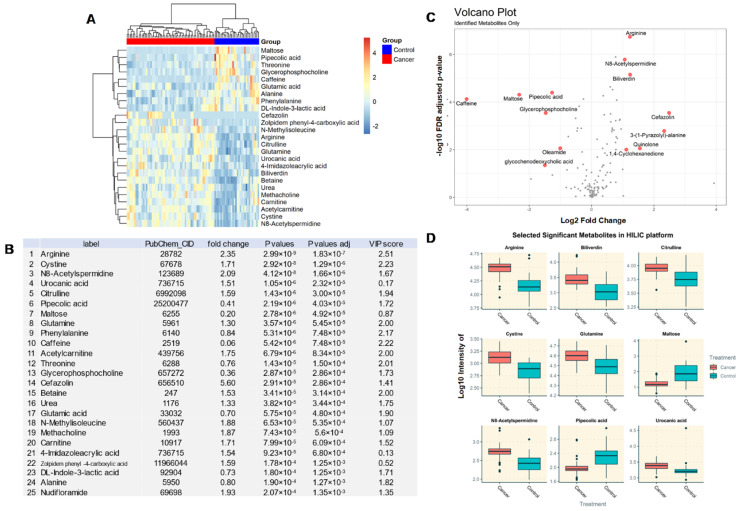
(**A**) Heatmap of identified metabolites in HILIC platform with the top 25 DEMs with the smallest Mann–Whitney U test *p*-values, comparing BC to control. Euclidean distance metric and Ward’s clustering method was used for the hierarchical clustering of samples and compounds. (**B**) The 25 identified metabolites are listed. Fold-changes are presented as the median average of cancer divided by control. False discovery rate-adjusted *p*-values using the Benjamini–Hochberg procedure are reported at the last column. VIP score for each compound is reported in Figure 3B (VIP > 1 highlighted as red). (**C**) Volcano plot visualizes the −log10 adjusted *p*-values and log2 fold-changes. Metabolites with adjusted *p*-values less than 0.05 and fold-change greater than 2-fold or less than 0.5 fold are highlighted and labeled. (**D**) Select example metabolite boxplots from the HILIC platform showing significant differences between BC and control.

Given that *different* analytical instruments for metabolomics profiling each have their own advantages and disadvantages, a smarter and innovative approach is to combine datasets *across* analytical *platforms* for more comprehensive coverage. A total of 139 DEMs was identified by HILIC-ESI-QTOF-MS/MS, of which the top 25 are shown in a heatmap (Figure 3A). These DEMs include arginine (fold-change of 2.35), carnitine (1.71), cystine (1.71), betaine (1.53), urea (1.33), glutamine (1.30), alanine (0.80), and maltose (0.20) (Figure 3B). Through an independent analysis (data not shown), a total of 23 known metabolites were shared across two different MS platforms with identical direction of expression changes. A volcano plot shows the representative DEMs whose expression were significantly altered in BC compared to controls *(*Figure 3C). The most significant DEMs are presented as box plots in Figure 3D. The expression levels of glutamine, citrulline, and urea were commonly upregulated in BC regardless of the analytical platform (Figure 2 and Figure 3).

### 3.4. ROC Curves of SVM.

**Figure 4:**
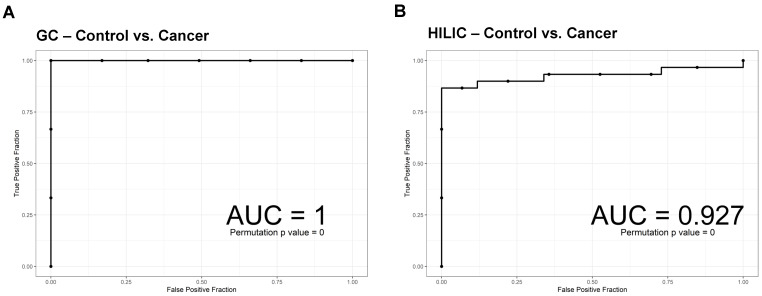
(**A**, **B**) Receiver operating characteristic (ROC) curves of support vector machine (SVM) with leave-one-out cross-validation of the area under the curve (AUC) using metabolites with adjusted *p*-values less than 0.05 in the GC (**A**) or HILIC platforms (**B**), respectively.

To predict the probability of a binary outcome, ROC curves of SVM were used with leave-one-out cross-validation. SVM was applied to distinguish cancer and control using significant compounds. To avoid the overfitting issue, the leave-one-out procedure was used when calculating the AUC of the ROC curve. The AUC was calculated using metabolites with adjusted *p*-values less than 0.05 from both the GC-TOF-MS and HILIC-ESI-QTOF-MS/MS platforms. The calculated AUCs were 1 and 0.927 for GC-TOF-MS and HILIC-ESI-QTOF-MS/MS, respectively (Figure 4). These data suggest that our model has a strong predicting power and reflects great differentiation capability. The permutation test returned a *p*-value less than 0.0001, showing that the model is validated and further guaranteed for non-overfitting. 

### 3.5. ChemRICH Plots of BC-Associated Metabolites

Next, we aimed to understand the biological meaning of the BC-specific metabolomes. Since biological interpretations of metabolic regulation in metabolomic datasets can be limited due to incomplete pathway definitions and enrichment statistics, ChemRICH, a newly developed pathway mapping tool, was applied [22]. Instead of traditional MSEA, which is determined by pre-defined compound cluster database, ChemRICH uses chemical ontologies and structure similarities to group metaoblites. *p*-values in ChemRICH are calculated using a self-contained Kolmogorov–Smirnov (KS) test and clusters metabolites into non-overlapping chemical groups rather than sparse biochemical knowledge annotations [26].

**Figure 5:**
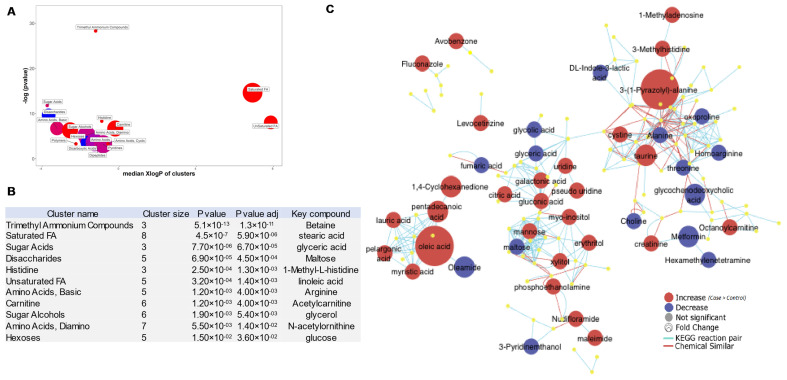
(**A**) ChemRICH analysis plot. Y-axis shows the most significantly altered clusters. Cluster color shows the proportion of increased or decreased metabolites compared to control (red = increased, blue = decreased, purple = mostly decreased). Cluster size indicates the number of compounds in each cluster. Chemical enrichment statistics were calculated by the Kolmogorov–Smirnov test. Only significantly different enrichment clusters (raw *p* < 0.05) are shown. (**B**) Statistics table for metabolite clusters. (**C**) MetaMapp metabolite network visualization. Red nodes indicate increased metabolites in BC compared to control, while the blue indicates a decrease. Node size indicates the magnitude of fold-change. Compounds are connected by KEGG reaction pair (blue line), and chemical similarity (red line).

The plot in Figure 5A shows the ChemRICH enrichment results with the most significantly impacted metabolite clusters of BC-specific DEMs (*p* < 0.05). The most significantly altered clusters were the trimethyl ammonium compounds (*p*-value = 5.1× 10^−13^; false discovery rate = 1.3× 10^−11^), which are located on the top of the plot y-axis in Figure 5A. The cluster colors give the proportion of increased or decreased compounds (red = increased, blue = decreased, purple = the most decreased). Several clusters, including the saturated fatty acid (FA) cluster (stearic acid as key component), unsaturated FA cluster (linoleic acid as key component), sugar alcohols (glycerol as key component), and carnitine (acetylcarnitine as key component), were found to be greatly increased in BC (Figure 5B). There were 11 metabolite clusters enriched with false discovery rate *p*-values less than 0.05. They include trimethyl ammonium compounds, saturated FA, sugar acids, disaccharides, histidine, unsaturated FA, basic amino acids, basic carnitine, sugar alcohols, diamino, and hexoses (Figure 5B). 

Next, to efficiently map and visualize the metabolomic data, MetaMapp, a tool for integrating information from biochemical pathways and chemical and mass spectral similarity was used [23]. Metamapp is able to map all detected metabolites into network graphs using the KEGG reactant pair database and Tanimoto chemical and National Institute of Standards and Technology (NIST) mass spectral similarity scores. Although Cytoscape (www.cytoscape.org) has been widely used for differential network visualizations and subnetwork identification, MetaMapp graphs in Cytoscape show clearer metabolic modularity and complete content visualization compared to conventional biochemical mapping approaches (http://metamapp.fiehnlab.ucdavis.edu) [27]. MetaMapp analysis revealed that several metabolites act as key players in the BC-specific metabolome. Red nodes reflect the metabolites found to be significantly upregulated in BC (*p*-value < 0.05) while those that are downregulated are shown as blue. The node sizes correlate with fold-change. Metabolites that were not found to be differentially regulated or “unknown” were left unlabeled for visual clarity. Red edges denote KEGG reactant pair links. Blue edges denote Tanimoto chemical similarity with T > 700. MetaMapp uses biochemical reaction pair information (Figure 5, blue lines) and chemical similarity (Figure 5, red lines) to create an overview of metabolic regulation. It clearly visible that saturated and unsaturated FAs are most upregulated, while disaccharides are the most downregulated. Oleic acid as well as 3-(1-Pyrazolyl)-alanine are two major central metabolites linked to many other metabolites upregulated in BC (Figure 5C).

### 3.6. Top 50 Metabolic Pathway-Associated DEMs Sets

**Figure 6:**
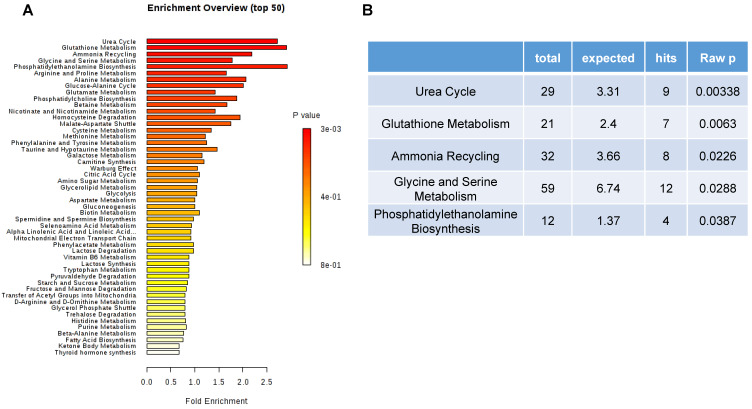
(**A**) Summary plot for over representation analysis (ORA) on metabolites with Mann–Whitney U test raw *p*-values less than 0.05. Top 50 metabolic pathway-associated metabolite sets are shown. (**B**) Five significant metabolic pathways with *p*-values less than 0.05.

Over representation analysis (ORA), as shown in Figure 6, was done to detect the impact of pathways, depending on the number of changed metabolites, and to test if a group of compounds was represented more than expected by chance. In the context of pathway analysis, compounds involved in a pathway are enriched and compared by random hits as tested. Detailed results from the pathway analysis are depicted in Figure 6A. Urea cycle, glutathione metabolism, ammonia recycling, glycine and serine metabolism, phosphatidylethanolamine biosynthesis, arginine and proline metabolism were found to be significant, with *p*-values less than 0.1 (Figure 6B).

## 4. Discussion

In this study, two independent analytic platforms combined with chemical similarity-based mapping and visualization tools revealed central clusters and network models in human BC tissue specimens. Both independent metabolomics’ profiling analyses suggested that glutamine metabolism, phosphatidylethanolamine biosynthesis, urea cycle, and ammonia recycling were significantly associated with BC. In addition, ChemRICH revealed trimethyl ammonium compounds, saturated FAs, and sugar acids as being the most significantly enriched metabolite clusters (false discovery rate adjusted *p*-values < 0.001) in BC compared to controls.

The data from this study showed that metabolites, including glutamine, citrulline, and urea, are upregulated in BC. Alterations in metabolism in BC has been reported to be characteristic of highly diverse BC. FA and glutamine [28] metabolisms are well-known in aggressive BC types, such as triple-negative BC (TNBC), whose expression of ASCT2/SLC1A5 (alanine, serine, cysteine-preferring transporter 2) was found to be increased. Enhanced glutamine metabolism is linked with protein and nucleotide synthesis in cancer [29,30]. Glutamine provides biosynthesis substrates, such as carbon and nitrogen, and acts as an energy resource through ATP biosynthesis. The inhibition of glutaminase exhibited antitumor activity in both the in vitro and in vivo BC models [31]. The metabolic signatures were linked to BC subtypes. The ER- subtype showed reprogrammed glutamine metabolism compared to the ER+ subtype [32,33]. Additionally, the ER- subtype is a preferential target for glutaminase inhibitors.

Our results showed increased citrulline in BC patients. However, the mechanisms behind this increase in citrulline remain unclear. Citrulline is a naturally occurring non-essential amino acid and an intermediate in the urea cycle. It is also a direct precursor of arginine, and its metabolic activity is mainly a result of its close link with arginine metabolism. There are three parallel metabolic transformations of citrulline, including arginine biosynthesis and the arginine–citrulline–nitric oxide (NO) cycle [34]. Citrulline is synthesized by ornithine transcarbamylase from ornithine and metabolized by argininosuccinate synthase in the urea cycle [35]. Alterations in the expression of urea cycle enzymes in BC [36] have revealed a revolutionary mechanism to maximize nitrogen incorporation into biomass [37]. The rewiring of urea cycle enzymes and the role of citrulline in cancer notes a new and exciting era for cancer metabolic studies. Further investigation into the functions of citrulline and its alterations is warranted and could lead to the identification of more effective therapeutic strategies against BC.

The data from this study also suggest that oleic and linoleic acid are enriched in BC, which is consistent with previous observations noting that changes in lipid metabolism are established hallmarks of BC [38]. Breast epithelial cells are embedded within a fat environment, which suggests that potential metabolites or substrates that are released during adipose lipolysis may be contributing to cancer progression [39]. We found that arginine, glutathione and sugar metabolism, and polyamines are altered in BC. Interestingly, arginine is involved in polyamine synthesis, which was found to also be implicated in BC [40,41,42]. Free fatty acids (FFAs) are an energy source and can induce activation of signal transduction pathways in BC cells [43]. However, the mechanisms through which altered FFA metabolism drives relapse has not been addressed. Oleic acid is one of the most common monounsaturated FFA in human adipocytes and other tissues [44]. Our analysis demonstrated that oleic acid is associated with BC compared to controls. Oleic acid prompts cell proliferation and migration in metastatic cancer through various pathways, including EGFR, AKT and NF-κB [45]. Linoleic acid is an essential and omega-6 polyunsaturated FA, which constitutes a major component of FAs in occidental diets. High fat diet intake has previously been shown to be associated with increased risk of BC [46]. Linoleic acid mediates a variety of cellular processes, including expression of plasminogen activator inhibitor-1 and cellular migration and invasion, and can induce an epithelial-to-mesenchymal transition-like process in BC [47]. Based on our metabolic observations, it could be postulated that the FFAs microenvironment might favor tumor progression, which provides novel potential targets for the chemoprevention of human cancer.

Current metabolomics biochemical knowledge/databases and mapping tools have limited coverage of detected metabolites. In order to resolve the shortcomings of mapping approaches and data visualization and to improve pathway analysis, we adopted ChemRICH and MetaMapp, which were recently developed in Fiehn laboratory. In this study, to better understand the metabolic signatures specific to BC and control groups, we performed ChemRICH and used Mann–Whitney U test *p*-values and median fold-changes to identify metabolites. To evaluate whether a metabolite cluster was represented more than expected by chance, the Kolmogorov–Smirnov test was performed on the identified clusters.

ChemRICH identifies enriched pathways using chemical similarity from medical subject headings and Tanimoto substructure chemical similarity coefficients. ChemRICH is useful for translating data obtained from clinical specimens, although it does not provide information regarding enzymes or diseases.

While conserving biochemical organization, the constructed MetaMapp-integrated network graph displays the key metabolites associated with BC. Significantly upregulated metabolites are denoted as red nodes and labeled with their respective BinBase names, while those downregulated are denoted as blue (*p*-value < 0.05). The node sizes are reflective of the amount of fold-change. Metabolites that were not differentially regulated in BC or unknown were left unlabeled for clarity. KEGG reactant pair links are reflected as red edges. Tanimoto chemical similarity, with T > 0.7, is reflected through blue edges. A MetaMapp-integrated network graph has several advantages. Firstly, the data are independent of the methodology used to acquire metabolomics profiles (i.e., MS or NMR). This allows for integration and visualization of data from different platforms. However, the only requirement is that all chemical structures are already encoded. Secondly, genomics is not a constraining factor when using MetaMapp. Detected metabolites can be mapped across studies or species; for instance, metabolites can be mapped from gut microbes to compounds that stem from mammalian enzymes. Lastly, the MetaMapp layout is dynamic and automatically updates based on the input of compound lists. As a result, MetaMapp graphs enable higher biochemical clarity despite having larger metabolic nodes. Since MetaMapp outputs are compatible with Cytoscape, next-generation metabolomic datasets with greater identified metabolites and integration of genomic and proteomic data can be visualized.

However, there are several shortcomings with MetaMapp. It cannot be used to compute flux or enzymatic reactions among metabolites. Although it is scalable to an extent, adding large numbers of nodes can lead to blurring. This was seen when we added a higher number of unknowns based on MS similarity. The visual clarity of MetaMapp can also decrease when statistical results are combined from further comparisons. As a result, multiple two-way graphs are recommended for displaying more complex biological studies.

We are aware there are some limitations in this study. First, subjects were not excluded based on menopausal status, so it is impossible to rule out hormone-related effects on data. Second, biospecimens were not obtained from the same patients or compared directly, although the aim of the current study was to explore potential BC biomarkers. Thus, we do not have detail clinical information such as the mutational status of collected tumours, which is a big limitation of this study. Further investigation into BC patients with different molecular characteristics will provide evidence suggesting potential prognostic and diagnostic tools for precision medicine. To further develop metabolic inhibitors as clinical regimens with existing therapies for BC patients, it is be critical to understand the heterogeneity in metabolism and targetable metabolic vulnerabilities in BC.

In summary, BC displays heterogeneous metabolic profiles similarly so unbiased global metabolomics profiling can reveal overlapped metabolic vulnerabilities in different BC types. The findings from this study provide another layer of evidence suggesting key metabolic players in BC. This can be further developed into therapeutic strategies to hinder or delay aggressive BC progression.

## Supplementary Materials

The following are available online at https://www.mdpi.com/2218-1989/10/4/170/s1, Table S1: GC-TOF-MS identified significant differentially expressed metabolites, Table S2: Metabolomics profiling using HILIC-ESI QTOF MS/MS identified compounds.
